# Supplementary figures and images for: Microfiber release from real soiled consumer laundry and the impact of fabric care products and washing conditions
Source: PLoS One. 2020 Jun 5;15(6):e0233332. doi: 10.1371/journal.pone.0233332 (PMC7274375; doi:10.1371/journal.pone.0233332)

**S1 Fig. Images of typical real wash loads**

| **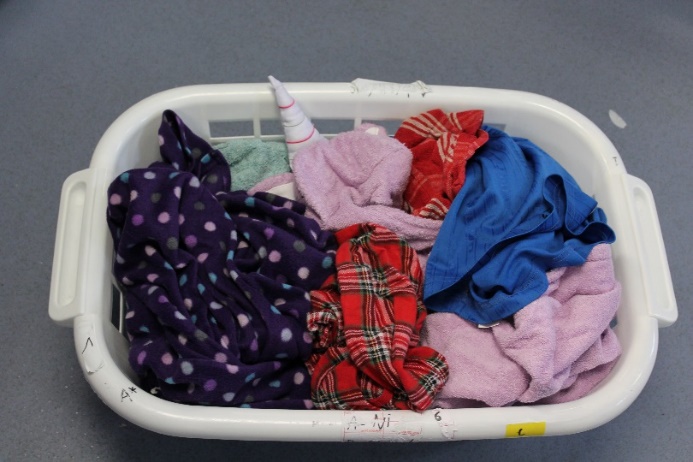** | **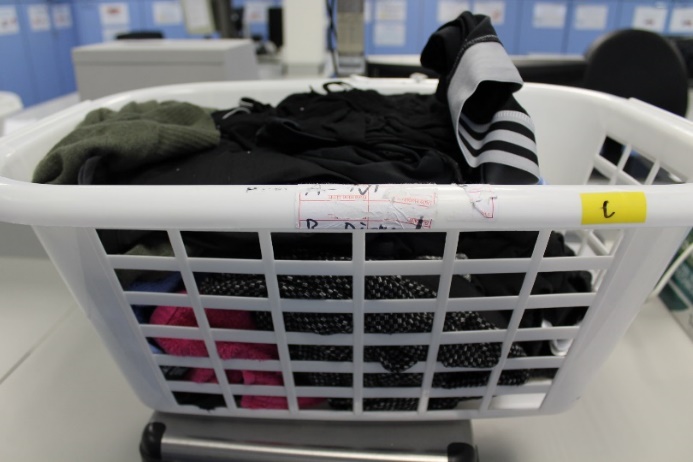** |
| --- | --- |
| **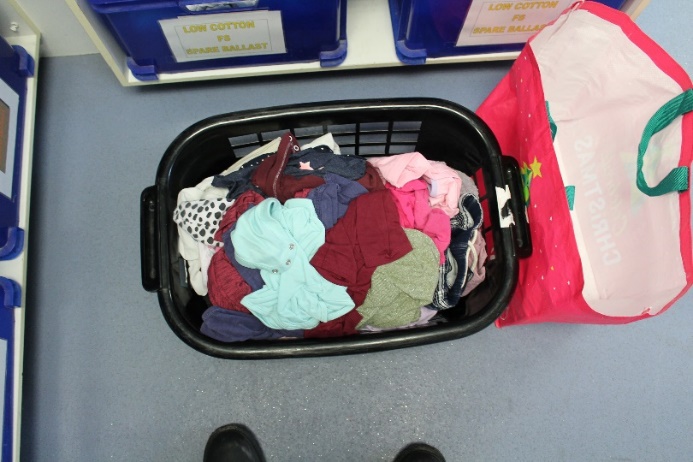** | **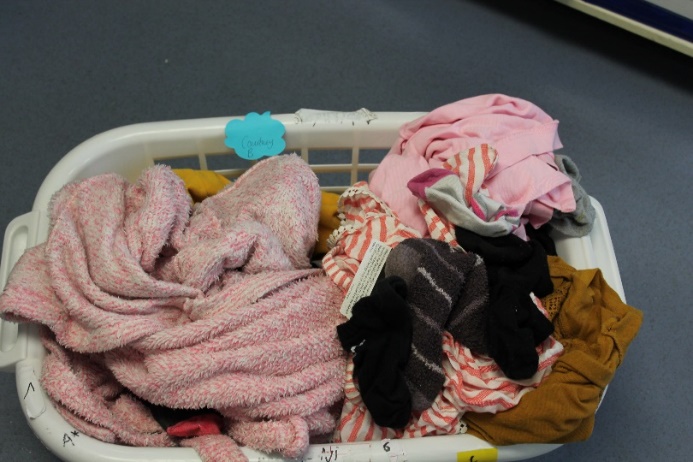** |
| **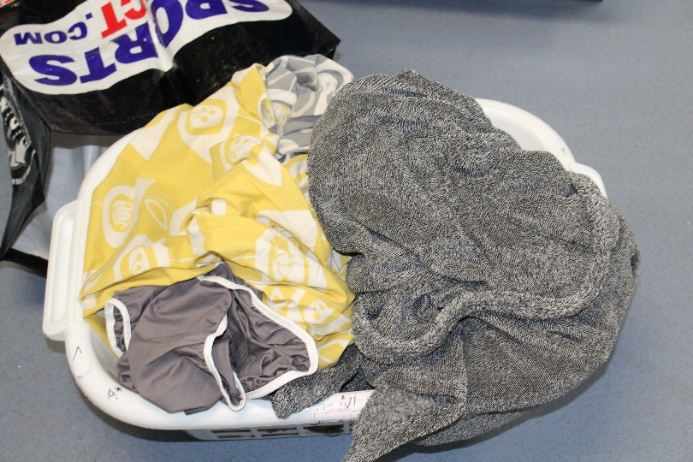** | **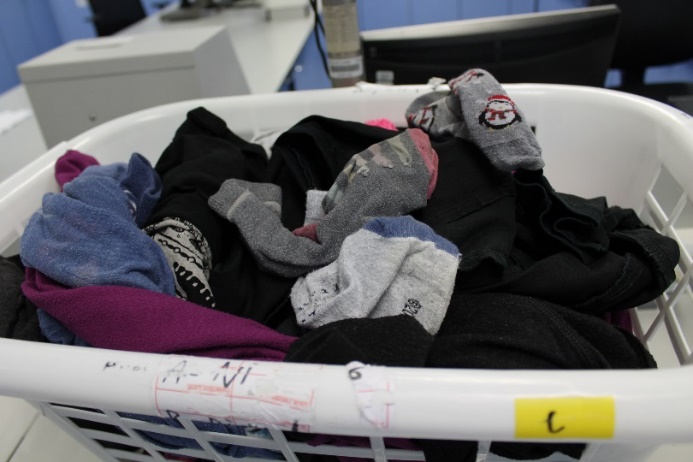** |
| **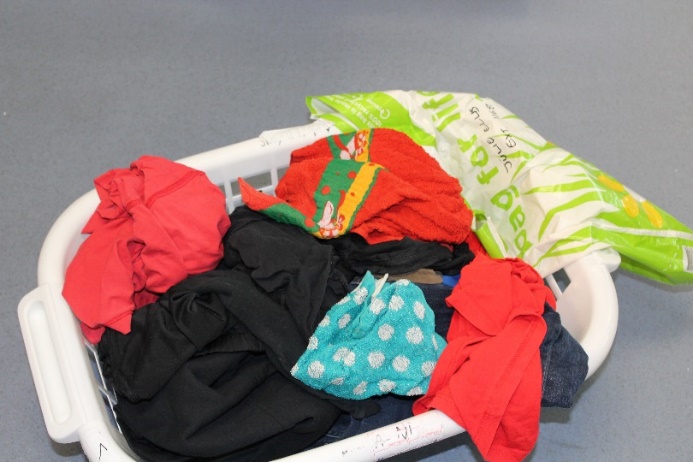** | **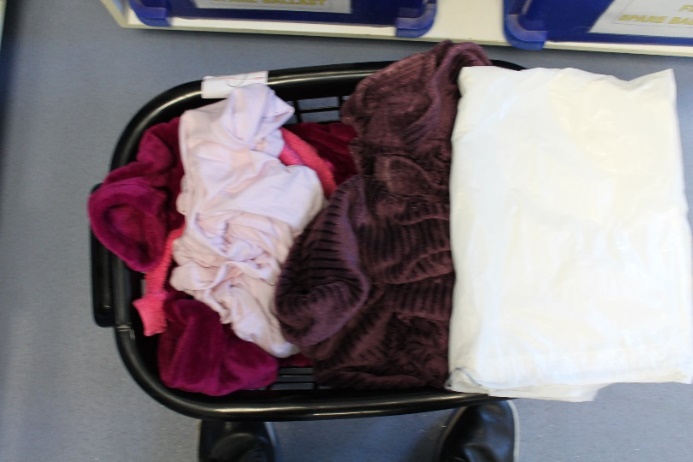** |
| **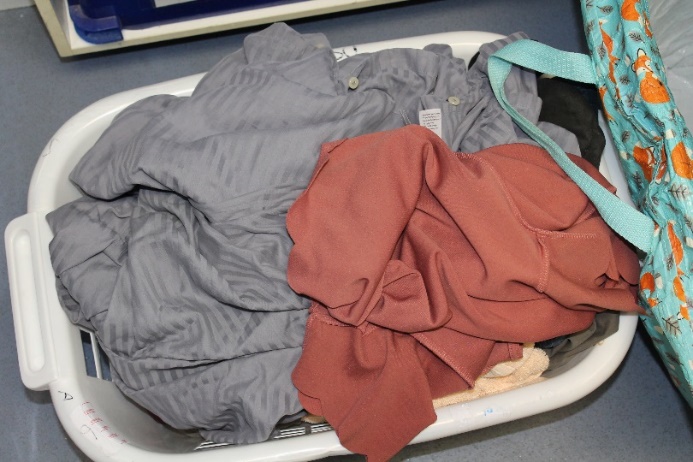** | **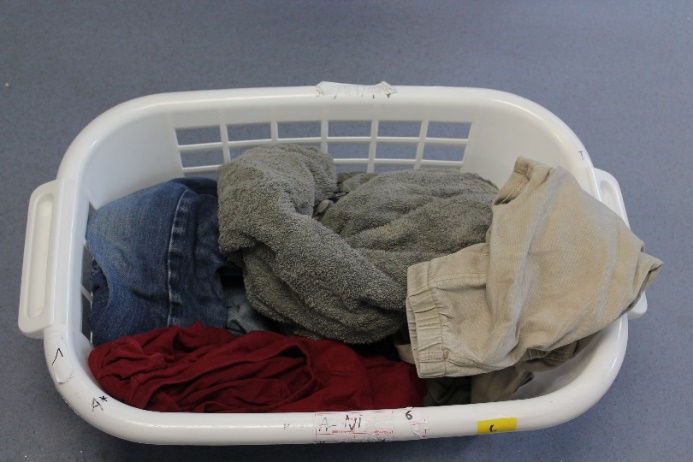** |
| **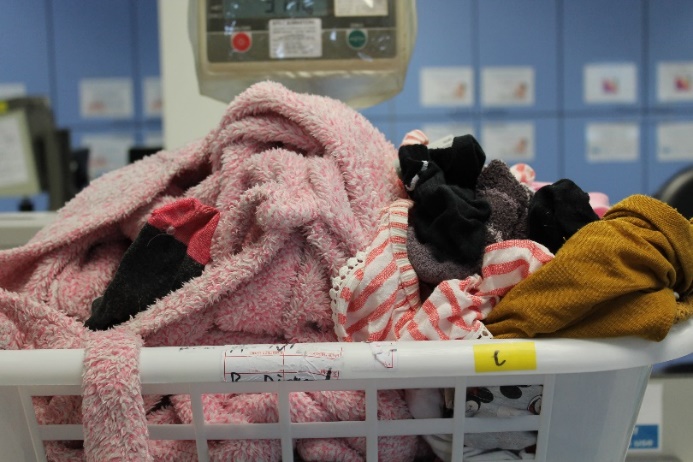** | **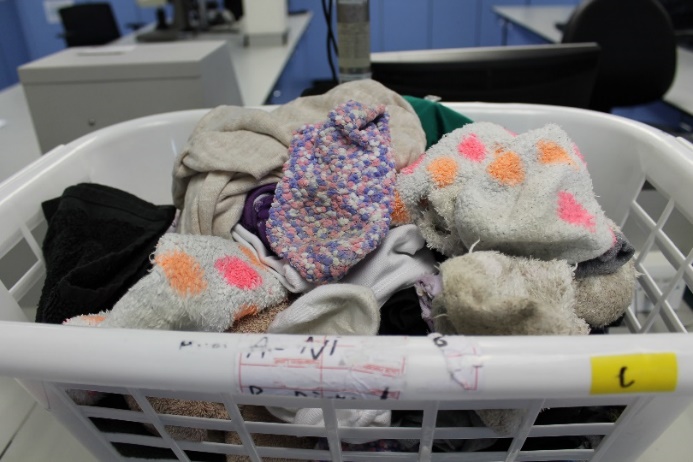** |
| **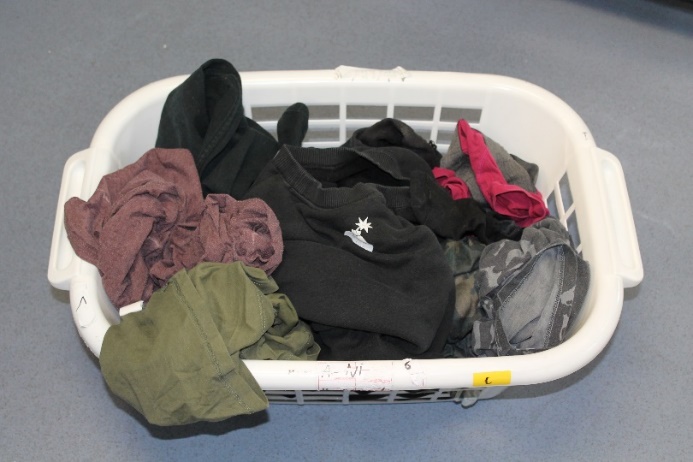** | **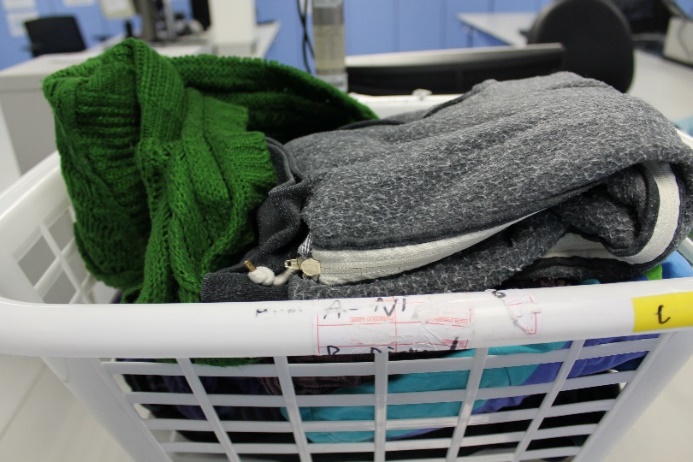** |
| **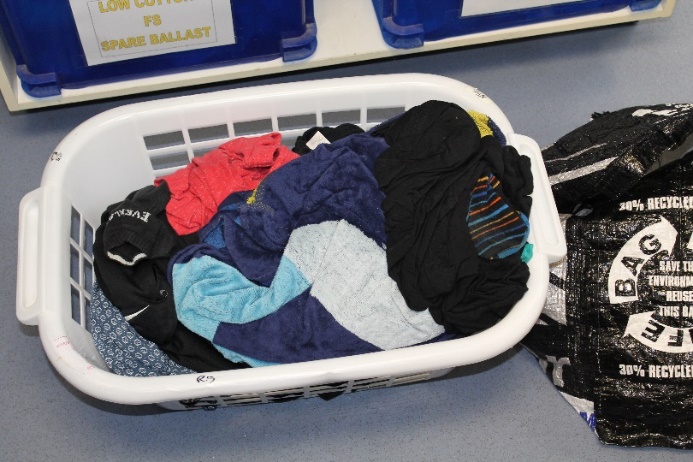** | **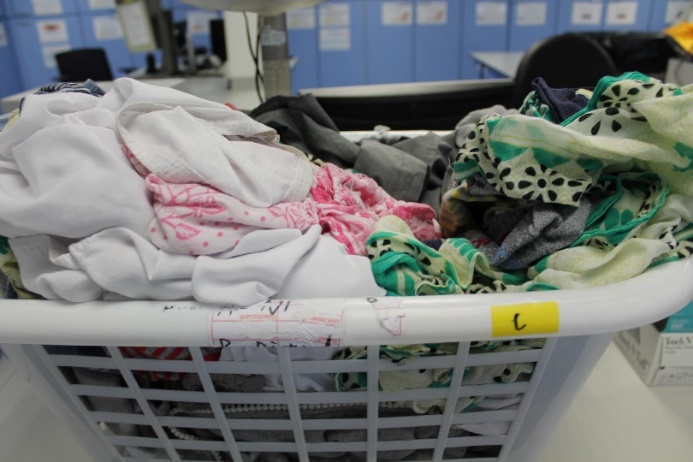** |
| **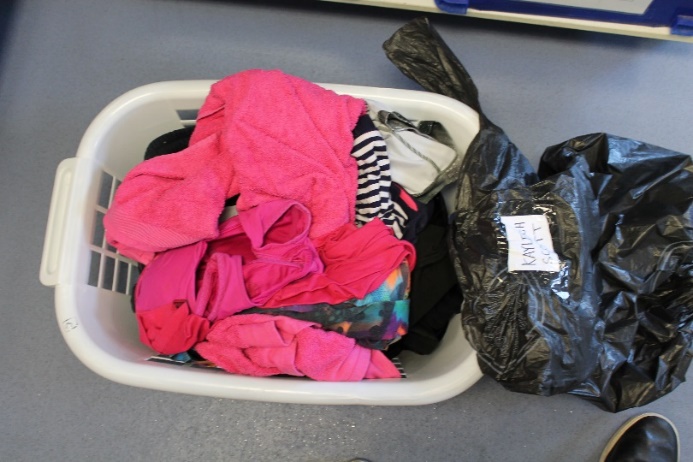** |  |

Supplement: S1 Fig — (DOCX) [file pone.0233332.s001.docx]

**S3 Fig. Light microscopy images of example filtered fibers.**

**
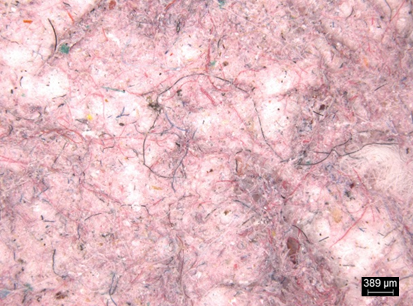
**

**
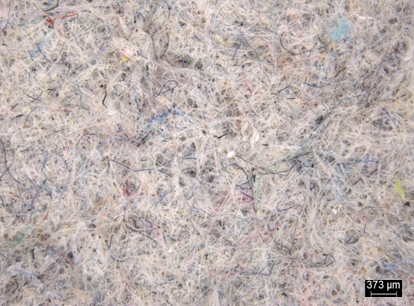
**

**
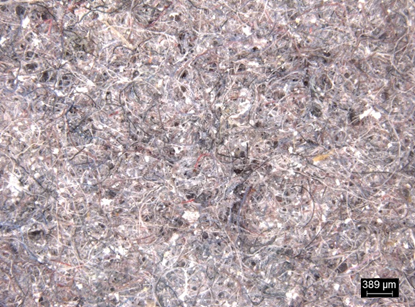
**

**
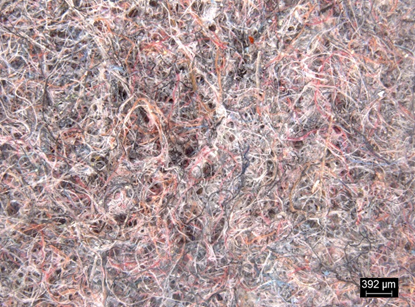
**

**
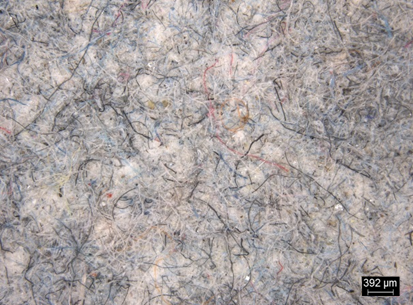
**

**
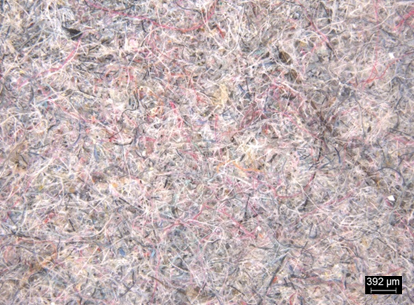
**

**
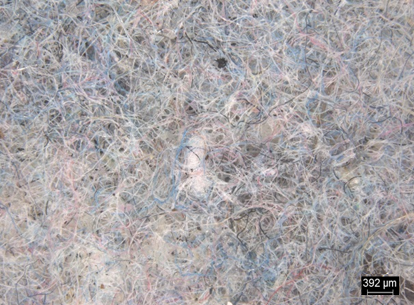
**

**
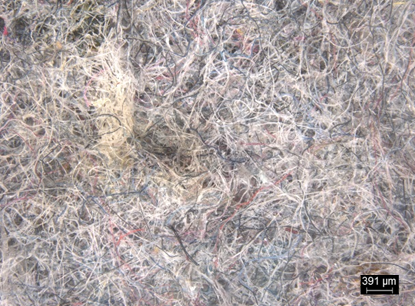
**

Supplement: S2 Fig — (DOCX) [file pone.0233332.s002.docx]

**S5 Fig. Boxplot of microfiber release from 79 European soiled consumer wash loads (n = 79).**

**
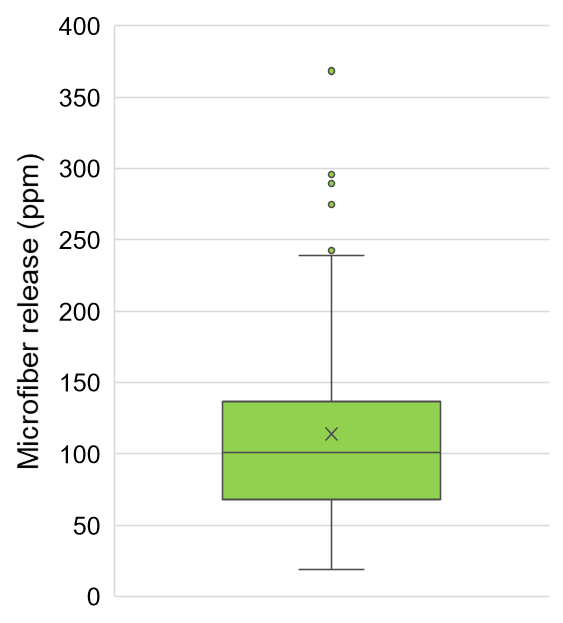
**

Supplement: S3 Fig — (DOCX) [file pone.0233332.s003.docx]
