# Supplementary material for: Microfiber release from real soiled consumer laundry and the impact of fabric care products and washing conditions
Source: PLoS One. 2020 Jun 5;15(6):e0233332. doi: 10.1371/journal.pone.0233332 (PMC7274375; doi:10.1371/journal.pone.0233332)
Supplement: S1 Table — (DOCX) [file pone.0233332.s004.docx]

**S2** **Table. List of whiteness monitor fabrics.**

| **Number** | **Manufacturer** | **Product Code** | **Weight**  **(g m^-2^)** | **Fiber Composition** |
| --- | --- | --- | --- | --- |
| W1 | Fruit of the Loom^®^ | 61082 | 135 | 100% cotton |
| W2 | Anvil^®^ | 980 | 150 | 100% cotton, |
| W3 | Gildan^®^ | 5000 | 170 | 100% cotton, |
| W4 | B&C^®^ | TU002 | 145 | 100% cotton, |
| W5 | Russell^®^ | R155M | 140 | 100% cotton, |
| W6 | Russell^®^ | ZT180M | 175 | 100% cotton, |
| W7 | Gildan^®^ | SUB42 | 169 | 100% polyester |
| W8 | Anvil^®^ | 6750 | 159 | 50% polyester, 25% cotton, 25% rayon |
| W9 | Fruit of the Loom^®^ | 61390 | 140 | 100% polyester |
| W10 | Kustom Kit^®^ | KK504 | 160 | 65% polyester, 35% cotton |
| W11 | Spiro^®^ | S253M | 160 | 100% polyester |
| W12 | Xpres^®^ | XP600 | 150 | 100% polyester |
| W13 | Russell^®^ | 165M | 155 | 65% polyester, 35% cotton |
| W14 | Tee Jays^®^ | TJ520 | 220 | 100% cotton |
| W15 | Spiro^®^ | S182M | 140 | 100% polyester |
| W16 | Tee Jays^®^ | TJ7020 | 160 | 95% polyester, 5% Spandex |
| W17 | Bella Canvas^®^ | CA3650 | 122 | 52% cotton, 48% polyester |
| W18 | Gildan^®^ | 42400 | 161 | 100% polyester |
