# Supplementary material for: Microfiber release from real soiled consumer laundry and the impact of fabric care products and washing conditions
Source: PLoS One. 2020 Jun 5;15(6):e0233332. doi: 10.1371/journal.pone.0233332 (PMC7274375; doi:10.1371/journal.pone.0233332)
Supplement: S2 Table — (DOCX) [file pone.0233332.s005.docx]

**S4** **Table. Load masses and microfiber masses for 79 European soiled consumer wash loads (n = 79).**

| **Reference** | **Load mass**  **(kg)** | **Microfiber mass (mg)** | **Microfiber release (ppm)*** |
| --- | --- | --- | --- |
| 1 | 2.55 | 317.2 | 124.41 |
| 2 | 2.95 | 180.8 | 61.30 |
| 3 | 1.96 | 305.7 | 155.97 |
| 4 | 2.57 | 164.7 | 64.07 |
| 5 | 2.04 | 751.4 | 368.32 |
| 6 | 3.09 | 243.0 | 78.65 |
| 7 | 2.80 | 237.7 | 84.89 |
| 8 | 2.40 | 160.3 | 66.81 |
| 9 | 2.72 | 280.1 | 102.98 |
| 10 | 2.09 | 431.2 | 206.31 |
| 11 | 2.69 | 119.5 | 44.41 |
| 12 | 2.52 | 148.2 | 58.79 |
| 13 | 2.04 | 602.8 | 295.51 |
| 14 | 2.18 | 380.2 | 174.39 |
| 15 | 5.42 | 193.6 | 35.72 |
| 16 | 1.75 | 317.9 | 181.68 |
| 17 | 3.60 | 311.6 | 86.54 |
| 18 | 2.41 | 311.1 | 129.11 |
| 19 | 2.58 | 318.3 | 123.39 |
| 20 | 4.34 | 439.2 | 101.19 |
| 21 | 2.07 | 599.3 | 289.50 |
| 22 | 2.62 | 293.4 | 112.00 |
| 23 | 3.30 | 166.8 | 50.55 |
| 24 | 3.54 | 261.4 | 73.85 |
| 25 | 2.77 | 662.0 | 238.99 |
| 26 | 3.19 | 305.3 | 95.71 |
| 27 | 4.46 | 190.5 | 42.72 |
| 28 | 5.28 | 98.3 | 18.62 |
| 29 | 4.27 | 227.4 | 53.26 |
| 30 | 2.19 | 235.6 | 107.56 |
| 31 | 2.48 | 197.6 | 79.68 |
| 32 | 2.73 | 318.9 | 116.82 |
| 33 | 4.11 | 501.0 | 121.91 |
| 34 | 5.92 | 441.1 | 74.51 |
| 35 | 2.23 | 247.3 | 110.91 |
| 36 | 1.52 | 341.7 | 224.83 |
| 37 | 3.19 | 230.0 | 72.11 |
| 38 | 4.06 | 176.5 | 43.47 |
| 39 | 4.93 | 255.2 | 51.76 |
| 40 | 4.58 | 250.8 | 54.76 |
| 41 | 3.16 | 574.6 | 181.84 |
| 42 | 3.06 | 358.3 | 117.08 |
| 43 | 1.30 | 244.6 | 188.13 |
| 44 | 2.02 | 187.7 | 92.90 |
| 45 | 2.07 | 373.7 | 180.55 |
| 46 | 2.38 | 389.1 | 163.49 |
| 47 | 2.50 | 434.4 | 173.77 |
| 48 | 2.23 | 398.6 | 178.75 |
| 49 | 4.61 | 313.9 | 68.09 |
| 50 | 2.98 | 350.8 | 117.72 |
| 51 | 3.06 | 205.7 | 67.23 |
| 52 | 1.66 | 175.7 | 105.82 |
| 53 | 3.43 | 342.3 | 99.81 |
| 54 | 3.47 | 274.2 | 79.02 |
| 55 | 4.54 | 341.3 | 75.18 |
| 56 | 3.26 | 790.8 | 242.57 |
| 57 | 5.06 | 305.3 | 60.33 |
| 58 | 4.17 | 235.2 | 56.41 |
| 59 | 2.67 | 385.8 | 144.48 |
| 60 | 2.56 | 280.9 | 109.71 |
| 61 | 4.35 | 400.6 | 92.09 |
| 62 | 2.78 | 205.6 | 73.95 |
| 63 | 4.62 | 541.5 | 117.21 |
| 64 | 1.60 | 439.9 | 274.94 |
| 65 | 2.96 | 318.5 | 107.61 |
| 66 | 2.62 | 229.9 | 87.73 |
| 67 | 3.40 | 417.3 | 122.75 |
| 68 | 4.30 | 338.3 | 78.68 |
| 69 | 2.66 | 387.0 | 145.48 |
| 70 | 3.57 | 288.6 | 80.85 |
| 71 | 3.27 | 183.1 | 55.99 |
| 72 | 2.39 | 327.0 | 136.84 |
| 73 | 3.37 | 294.7 | 87.46 |
| 74 | 3.32 | 345.5 | 104.05 |
| 75 | 3.23 | 359.0 | 111.15 |
| 76 | 3.69 | 131.5 | 35.63 |
| 77 | 5.40 | 196.3 | 36.35 |
| 78 | 2.09 | 218.5 | 104.54 |
| 79 | 3.45 | 242.3 | 70.23 |
| **Mean** | **3.13** | **317.0** | **113.98** |
| **Standard Deviation** | **1.02** | **136.8** | **66.79** |

***Microfiber release (ppm) = Microfiber mass (mg) / Load mass (kg)**
