# Supplementary material for: Microfiber release from real soiled consumer laundry and the impact of fabric care products and washing conditions
Source: PLoS One. 2020 Jun 5;15(6):e0233332. doi: 10.1371/journal.pone.0233332 (PMC7274375; doi:10.1371/journal.pone.0233332)
Supplement: S3 Table — (DOCX) [file pone.0233332.s006.docx]

**S6 Table. Digestion of fibers**

| **Sample ID** | **% Synthetic** | **% Natural** |
| --- | --- | --- |
| 2 | 2.70 | 97.30 |
| 3 | 2.72 | 97.28 |
| 4 | 1.04 | 98.96 |
| 6 | 5.29 | 94.71 |
| 11 | 2.40 | 97.60 |
| 15 | 1.45 | 98.55 |
| 20 | 2.07 | 97.93 |
| 24 | 5.99 | 94.01 |
| 27 | 8.29 | 91.71 |
| 31 | 5.94 | 94.06 |
| 36 | 4.54 | 95.46 |
| 41 | 2.12 | 97.88 |
| 42 | 1.79 | 98.21 |
| 43 | 2.82 | 97.18 |
| 45 | 4.91 | 95.09 |
| 46 | 5.42 | 94.58 |
| 47 | 4.34 | 95.66 |
| 48 | 1.54 | 98.46 |
| 49 | 2.42 | 97.58 |
| 53 | 4.48 | 95.52 |
| 58 | 5.49 | 94.51 |
| 62 | 9.96 | 90.04 |
| 65 | 6.33 | 93.67 |
| 69 | 2.70 | 97.30 |
| 78 | 5.07 | 94.93 |
| **Mean** | **4.07** | **95.93** |
| **Standard Deviation** | **2.24** | **2.24** |
