# Supplementary material for: Microfiber release from real soiled consumer laundry and the impact of fabric care products and washing conditions
Source: PLoS One. 2020 Jun 5;15(6):e0233332. doi: 10.1371/journal.pone.0233332 (PMC7274375; doi:10.1371/journal.pone.0233332)
Supplement: S4 Table — (DOCX) [file pone.0233332.s007.docx]

**S7** **Table. Microfiber release from soiled consumer wash loads in a 40°C cycle (n = 19) with Cold Express cycle (n = 19).**

| **40°C cycle** | | | |
| --- | --- | --- | --- |
| **Reference** | **Load mass**  **(kg)** | **Microfiber mass**  **(mg)** | **Microfiber release (ppm)*** |
| 1 | 3.39 | 475.6 | 140.30 |
| 2 | 3.16 | 321.7 | 101.80 |
| 3 | 2.19 | 377.7 | 172.47 |
| 4 | 2.84 | 316.2 | 111.34 |
| 5 | 3.53 | 258.3 | 73.17 |
| 6 | 1.90 | 477.9 | 251.53 |
| 7 | 1.56 | 337.7 | 216.47 |
| 8 | 2.28 | 401.9 | 176.29 |
| 9 | 2.52 | 474.2 | 188.19 |
| 10 | 3.65 | 483.5 | 132.48 |
| 11 | 2.18 | 244.6 | 112.22 |
| 12 | 2.44 | 463.5 | 189.97 |
| 13 | 1.66 | 720.5 | 434.06 |
| 14 | 3.23 | 369.3 | 114.34 |
| 15 | 1.59 | 530.6 | 333.72 |
| 16 | 3.06 | 356.9 | 116.62 |
| 17 | 4.36 | 930.1 | 213.34 |
| 18 | 3.10 | 487.1 | 157.13 |
| 19 | 2.39 | 513.8 | 215.00 |
| **Mean** | **2.69** | **449.5** | **181.60** |
| **Standard Deviation** | **0.77** | **161.2** | **87.14** |
| **Cold Express cycle** | | | |
| **Reference** | **Load mass**  **(kg)** | **Microfiber mass**  **(mg)** | **Microfiber release**  **(ppm)*** |
| 1 | 2.19 | 219.8 | 100.38 |
| 2 | 3.43 | 364.8 | 106.37 |
| 3 | 3.31 | 563.7 | 170.31 |
| 4 | 1.64 | 308.8 | 188.31 |
| 5 | 2.59 | 222.1 | 85.74 |
| 6 | 3.15 | 282.1 | 89.54 |
| 7 | 3.59 | 398.8 | 111.10 |
| 8 | 2.02 | 367.1 | 181.74 |
| 9 | 2.36 | 268.5 | 113.75 |
| 10 | 1.98 | 303.6 | 153.35 |
| 11 | 2.61 | 342.0 | 131.05 |
| 12 | 1.84 | 171.9 | 93.44 |
| 13 | 3.98 | 428.5 | 107.65 |
| 14 | 3.14 | 426.5 | 135.82 |
| 15 | 2.47 | 480.5 | 194.52 |
| 16 | 1.56 | 344.9 | 221.09 |
| 17 | 4.15 | 306.5 | 73.86 |
| 18 | 2.43 | 226.2 | 93.08 |
| 19 | 2.62 | 287.5 | 109.74 |
| **Mean** | **2.69** | **332.3** | **129.52** |
| **Standard Deviation** | **0.76** | **98.0** | **42.92** |

***Microfiber release (ppm) = Microfiber mass (mg) / Load mass (kg)**
