# Supplementary material for: Microfiber release from real soiled consumer laundry and the impact of fabric care products and washing conditions
Source: PLoS One. 2020 Jun 5;15(6):e0233332. doi: 10.1371/journal.pone.0233332 (PMC7274375; doi:10.1371/journal.pone.0233332)
Supplement: S5 Table — (DOCX) [file pone.0233332.s008.docx]

**S8** **Table. Comparison of whiteness loss (∆E_2000_) from 40°C (n=19) with Cold Express cycle (n=19) on 18 fabric types.**

|  | **40°C cycle - Replicates** | | | | | | | | | | | | | | | | | | |  |  |
| --- | --- | --- | --- | --- | --- | --- | --- | --- | --- | --- | --- | --- | --- | --- | --- | --- | --- | --- | --- | --- | --- |
| **Tracer** | 1 | 2 | 3 | 4 | 5 | 6 | 7 | 8 | 9 | 10 | 11 | 12 | 13 | 14 | 15 | 16 | 17 | 18 | 19 | **Mean** | **S.D.** |
| W1 | 2.24 | 7.03 | 6.73 | 7.26 | 1.18 | 0.62 | 1.24 | 4.44 | 1.41 | 1.53 | 1.50 | 1.60 | 1.59 | 2.81 | 1.09 | 1.18 | 2.88 | 3.12 | 5.85 | 2.91 | 2.22 |
| W2 | 0.58 | 0.29 | 0.42 | 1.25 | 2.20 | 0.73 | 2.08 | 5.09 | 0.60 | 2.25 | 3.01 | 1.35 | 1.61 | 3.13 | 1.01 | 0.67 | 2.22 | 3.48 | 4.66 | 1.93 | 1.42 |
| W3 | 3.10 | 2.13 | 2.37 | 2.61 | 1.28 | 0.61 | 4.23 | 3.76 | 0.70 | 1.12 | 1.05 | 0.69 | 1.47 | 2.67 | 0.67 | 0.56 | 1.79 | 1.68 | 4.15 | 1.93 | 1.22 |
| W4 | 5.20 | 0.86 | 0.99 | 2.14 | 1.30 | 0.68 | 2.14 | 2.98 | 0.88 | 1.62 | 1.93 | 2.98 | 0.95 | 2.97 | 0.90 | 0.88 | 2.51 | 3.72 | 4.83 | 2.13 | 1.37 |
| W5 | 4.46 | 2.25 | 3.64 | 2.16 | 1.43 | 0.65 | 1.83 | 2.85 | 0.55 | 0.98 | 2.57 | 0.32 | 2.72 | 3.03 | 0.65 | 0.30 | 1.88 | 2.44 | 6.36 | 2.16 | 1.55 |
| W6 | 0.90 | 0.75 | 0.93 | 1.76 | 1.37 | 0.90 | 2.33 | 3.00 | 1.36 | 1.83 | 1.83 | 0.36 | 1.72 | 4.21 | 1.91 | 0.97 | 2.48 | 2.81 | 7.00 | 2.02 | 1.52 |
| W7 | 0.75 | 0.91 | 0.26 | 1.24 | 1.74 | 0.45 | 0.70 | 3.80 | 0.33 | 0.83 | 0.92 | 0.43 | 0.83 | 2.49 | 0.64 | 0.21 | 1.08 | 0.85 | 3.57 | 1.16 | 1.04 |
| W8 | 2.21 | 1.81 | 2.23 | 1.64 | 4.37 | 0.72 | 0.77 | 4.26 | 2.02 | 1.64 | 1.46 | 0.64 | 1.57 | 4.02 | 1.66 | 0.72 | 1.81 | 1.58 | 4.05 | 2.06 | 1.22 |
| W9 | 0.74 | 1.02 | 0.65 | 0.69 | 0.67 | 0.84 | 0.54 | 2.57 | 0.66 | 0.41 | 0.56 | 1.51 | 0.34 | 1.32 | 0.39 | 0.89 | 0.38 | 0.32 | 2.72 | 0.91 | 0.69 |
| W10 | 2.67 | 1.80 | 0.86 | 2.14 | 1.16 | 0.93 | 0.27 | 3.51 | 0.33 | 0.55 | 0.32 | 1.00 | 1.05 | 2.41 | 0.49 | 0.45 | 0.68 | 1.11 | 3.64 | 1.34 | 1.06 |
| W11 | 1.39 | 0.96 | 0.67 | 0.93 | 1.04 | 0.34 | 0.70 | 4.90 | 0.77 | 1.14 | 1.34 | 0.44 | 1.58 | 2.96 | 0.75 | 0.56 | 1.21 | 1.22 | 4.19 | 1.43 | 1.24 |
| W12 | 1.48 | 1.34 | 0.63 | 0.97 | 0.46 | 0.27 | 0.52 | 3.25 | 0.58 | 0.69 | 0.95 | 0.69 | 1.04 | 2.26 | 0.52 | 0.60 | 0.90 | 1.02 | 3.12 | 1.12 | 0.86 |
| W13 | 1.49 | 0.35 | 0.40 | 1.47 | 1.07 | 0.96 | 0.60 | 2.65 | 0.50 | 0.21 | 0.60 | 0.81 | 0.88 | 2.37 | 0.66 | 0.46 | 0.89 | 1.03 | 3.04 | 1.08 | 0.80 |
| W14 | 1.74 | 2.43 | 1.75 | 3.37 | 1.97 | 1.35 | 2.92 | 7.04 | 2.67 | 3.34 | 3.10 | 0.95 | 1.97 | 5.67 | 1.59 | 1.43 | 4.32 | 6.68 | 6.90 | 3.22 | 1.98 |
| W15 | 2.69 | 3.25 | 1.31 | 0.28 | 0.54 | 0.31 | 0.44 | 3.43 | 0.37 | 0.99 | 4.71 | 0.44 | 1.18 | 2.70 | 0.55 | 0.33 | 1.21 | 1.08 | 3.64 | 1.55 | 1.39 |
| W16 | 3.42 | 0.64 | 1.79 | 1.33 | 0.68 | 0.51 | 0.88 | 5.99 | 1.14 | 1.56 | 1.40 | 1.26 | 3.09 | 5.01 | 1.64 | 0.66 | 1.94 | 1.84 | 5.75 | 2.13 | 1.72 |
| W17 | 1.69 | 1.13 | 0.73 | 0.71 | 0.66 | 0.35 | 0.28 | 2.48 | 0.44 | 0.53 | 0.26 | 0.28 | 1.19 | 1.96 | 0.59 | 0.47 | 1.61 | 1.20 | 3.96 | 1.08 | 0.94 |
| W18 | 2.41 | 1.42 | 0.85 | 1.25 | 0.41 | 0.90 | 0.39 | 2.57 | 0.40 | 0.64 | 0.43 | 0.47 | 0.84 | 1.93 | 0.26 | 0.29 | 1.03 | 0.54 | 2.60 | 1.03 | 0.79 |
| **Mean of Means:** | | | | | | | | | | | | | | | | | | | | **1.73** |  |
|  | **Cold Express cycle - Replicates** | | | | | | | | | | | | | | | | | | |  |  |
| **Tracer** | 1 | 2 | 3 | 4 | 5 | 6 | 7 | 8 | 9 | 10 | 11 | 12 | 13 | 14 | 15 | 16 | 17 | 18 | 19 | **Mean** | **S.D.** |
| W1 | 7.59 | 0.51 | 5.94 | 5.03 | 0.94 | 0.67 | 0.77 | 1.35 | 1.33 | 1.07 | 1.03 | 0.88 | 0.99 | 0.82 | 0.50 | 0.91 | 0.59 | 0.74 | 0.51 | 1.69 | 2.06 |
| W2 | 2.13 | 0.55 | 0.86 | 0.21 | 0.91 | 1.37 | 0.79 | 1.54 | 1.43 | 1.06 | 1.67 | 2.32 | 1.10 | 0.93 | 0.72 | 0.65 | 0.76 | 0.28 | 0.98 | 1.07 | 0.56 |
| W3 | 2.10 | 6.35 | 1.47 | 0.23 | 0.38 | 0.66 | 0.41 | 0.40 | 0.85 | 0.36 | 0.54 | 0.43 | 0.50 | 0.71 | 0.47 | 1.40 | 0.33 | 0.45 | 0.60 | 0.98 | 1.38 |
| W4 | 1.49 | 0.98 | 0.31 | 1.60 | 0.28 | 0.75 | 0.73 | 1.35 | 1.75 | 1.66 | 1.43 | 0.86 | 0.87 | 0.57 | 0.46 | 0.78 | 0.57 | 0.24 | 0.27 | 0.89 | 0.51 |
| W5 | 2.37 | 1.84 | 1.06 | 2.06 | 0.69 | 0.22 | 0.47 | 0.44 | 0.82 | 0.27 | 1.36 | 0.31 | 0.69 | 0.48 | 0.26 | 0.55 | 0.37 | 0.57 | 0.43 | 0.80 | 0.64 |
| W6 | 1.08 | 5.01 | 0.46 | 0.87 | 0.72 | 1.17 | 0.54 | 1.18 | 1.47 | 1.31 | 1.16 | 1.60 | 1.02 | 0.53 | 0.73 | 0.69 | 0.47 | 0.91 | 0.38 | 1.12 | 1.01 |
| W7 | 0.78 | 1.90 | 1.43 | 0.19 | 0.54 | 1.14 | 0.59 | 0.44 | 0.48 | 0.94 | 0.82 | 0.44 | 0.76 | 0.90 | 0.20 | 0.59 | 0.28 | 0.54 | 0.31 | 0.70 | 0.43 |
| W8 | 3.75 | 2.90 | 2.96 | 0.84 | 1.30 | 1.02 | 1.10 | 0.75 | 2.24 | 2.20 | 0.91 | 0.73 | 0.79 | 1.50 | 0.88 | 0.48 | 0.77 | 0.41 | 0.28 | 1.36 | 0.98 |
| W9 | 2.24 | 0.40 | 1.50 | 0.85 | 0.85 | 0.97 | 1.00 | 1.06 | 0.68 | 0.70 | 0.70 | 1.00 | 0.74 | 0.64 | 1.00 | 0.80 | 0.73 | 1.10 | 1.16 | 0.95 | 0.39 |
| W10 | 9.40 | 2.56 | 1.84 | 1.55 | 0.35 | 0.47 | 0.70 | 0.74 | 0.73 | 0.66 | 0.45 | 0.95 | 1.00 | 0.34 | 0.56 | 0.52 | 0.41 | 0.69 | 0.94 | 1.31 | 2.04 |
| W11 | 3.38 | 1.10 | 0.36 | 0.43 | 0.62 | 0.51 | 0.27 | 0.73 | 0.74 | 0.79 | 0.74 | 0.27 | 1.34 | 0.66 | 0.43 | 0.68 | 0.77 | 0.18 | 0.65 | 0.77 | 0.69 |
| W12 | 3.24 | 1.86 | 0.61 | 1.20 | 0.57 | 0.55 | 0.59 | 0.37 | 0.35 | 0.33 | 0.60 | 0.52 | 0.63 | 0.31 | 0.49 | 0.30 | 0.32 | 0.67 | 0.21 | 0.72 | 0.72 |
| W13 | 2.30 | 0.61 | 1.88 | 0.23 | 0.94 | 0.84 | 1.36 | 0.33 | 0.33 | 0.42 | 0.42 | 1.24 | 0.62 | 0.65 | 0.67 | 0.76 | 0.98 | 0.92 | 0.75 | 0.85 | 0.53 |
| W14 | 3.27 | 1.32 | 1.47 | 1.26 | 0.67 | 0.92 | 1.05 | 1.09 | 2.13 | 1.37 | 1.83 | 0.94 | 1.18 | 1.43 | 0.95 | 1.02 | 0.92 | 1.35 | 1.43 | 1.35 | 0.58 |
| W15 | 5.07 | 1.36 | 0.66 | 1.17 | 0.33 | 0.55 | 2.88 | 0.32 | 0.65 | 0.57 | 0.68 | 0.32 | 1.09 | 0.61 | 0.39 | 2.91 | 0.59 | 0.17 | 0.25 | 1.08 | 1.24 |
| W16 | 2.45 | 1.01 | 1.79 | 1.84 | 1.62 | 1.26 | 0.33 | 0.81 | 0.94 | 1.39 | 1.22 | 0.74 | 1.13 | 1.12 | 0.55 | 0.64 | 0.68 | 0.56 | 0.60 | 1.09 | 0.54 |
| W17 | 1.76 | 0.96 | 0.19 | 0.64 | 0.25 | 0.18 | 0.74 | 0.21 | 0.60 | 0.29 | 0.27 | 0.70 | 0.53 | 1.71 | 0.72 | 0.55 | 0.44 | 0.80 | 0.30 | 0.62 | 0.46 |
| W18 | 0.69 | 0.38 | 2.51 | 0.47 | 0.34 | 0.29 | 0.85 | 0.65 | 0.75 | 1.44 | 0.62 | 0.81 | 0.27 | 0.25 | 0.24 | 0.45 | 0.62 | 0.22 | 0.39 | 0.64 | 0.54 |
| **Mean of Means:** | | | | | | | | | | | | | | | | | | | | **1.00** |  |
