# Supplementary material for: Microfiber release from real soiled consumer laundry and the impact of fabric care products and washing conditions
Source: PLoS One. 2020 Jun 5;15(6):e0233332. doi: 10.1371/journal.pone.0233332 (PMC7274375; doi:10.1371/journal.pone.0233332)
Supplement: S6 Table — (DOCX) [file pone.0233332.s009.docx]

**S9** **Table. Impact of using European detergent pod on microfiber release from polyester fleece (n = 24).**

| **No Detergent** | | | |
| --- | --- | --- | --- |
|  | **Load mass**  **(kg)** | **Microfiber mass**  **(mg)** | **Microfiber release (ppm)*** |
| Cycle 1 – Load 1 | 1.750 | 195.21 | 111.55 |
| Cycle 1 – Load 2 | 1.740 | 122.22 | 70.24 |
| Cycle 1 – Load 3 | 1.750 | 92.22 | 52.70 |
| Cycle 1 – Load 4 | 1.760 | 79.23 | 45.02 |
| **Cycle 1 - Mean** | **1.750** | **122.22** | **69.88** |
| **Cycle 1 – Std Dev** | **0.008** | **51.88** | **29.72** |
| Cycle 4 – Load 1 | 1.750 | 72.14 | 41.22 |
| Cycle 4 – Load 2 | 1.740 | 68.08 | 39.12 |
| Cycle 4 – Load 3 | 1.750 | 71.04 | 40.60 |
| Cycle 4 – Load 4 | 1.760 | 59.29 | 33.69 |
| **Cycle 4 - Mean** | **1.750** | **67.64** | **38.66** |
| **Cycle 4 – Std Dev** | **0.008** | **5.83** | **3.43** |
| Cycle 8 – Load 1 | 1.750 | 49.38 | 28.22 |
| Cycle 8 – Load 2 | 1.740 | 63.90 | 36.72 |
| Cycle 8 – Load 3 | 1.750 | 27.43 | 15.68 |
| Cycle 8 – Load 4 | 1.760 | 30.61 | 17.39 |
| **Cycle 8 - Mean** | **1.750** | **42.83** | **24.50** |
| **Cycle 8 – Std Dev** | **0.008** | **17.06** | **9.86** |
| **European Pod** | | | |
|  | **Load mass**  **(kg)** | **Microfiber mass**  **(mg)** | **Microfiber release**  **(ppm)*** |
| Cycle 1 – Load 1 | 1.750 | 115.96 | 66.26 |
| Cycle 1 – Load 2 | 1.760 | 123.74 | 70.31 |
| Cycle 1 – Load 3 | 1.760 | 147.12 | 83.59 |
| Cycle 1 – Load 4 | 1.770 | 108.47 | 61.28 |
| **Cycle 1 - Mean** | **1.760** | **123.82** | **70.36** |
| **Cycle 1 – Std Dev** | **0.008** | **16.73** | **9.56** |
| Cycle 4 – Load 1 | 1.750 | 91.60 | 52.34 |
| Cycle 4 – Load 2 | 1.760 | 96.40 | 54.77 |
| Cycle 4 – Load 3 | 1.760 | 55.39 | 31.47 |
| Cycle 4 – Load 4 | 1.770 | 94.15 | 53.19 |
| **Cycle 4 - Mean** | **1.760** | **84.38** | **47.94** |
| **Cycle 4 – Std Dev** | **0.008** | **19.43** | **11.03** |
| Cycle 8 – Load 1 | 1.750 | 57.44 | 32.82 |
| Cycle 8 – Load 2 | 1.760 | 67.95 | 38.61 |
| Cycle 8 – Load 3 | 1.760 | 33.12 | 18.82 |
| Cycle 8 – Load 4 | 1.770 | 37.92 | 21.42 |
| **Cycle 8 - Mean** | **1.760** | **49.11** | **27.92** |
| **Cycle 8 – Std Dev** | **0.008** | **16.38** | **9.37** |

***Microfiber release (ppm) = Microfiber mass (mg) / Load mass (kg)**
