# Supplementary material for: Microfiber release from real soiled consumer laundry and the impact of fabric care products and washing conditions
Source: PLoS One. 2020 Jun 5;15(6):e0233332. doi: 10.1371/journal.pone.0233332 (PMC7274375; doi:10.1371/journal.pone.0233332)
Supplement: S7 Table — (DOCX) [file pone.0233332.s010.docx]

**S10** **Table. Impact of fabric softener on microfiber release from polyester fleece: European conditions (n = 36).**

| **European Pod** | | | |
| --- | --- | --- | --- |
|  | **Load mass**  **(kg)** | **Microfiber mass**  **(mg)** | **Microfiber release (ppm)*** |
| Cycle 1 – Load 1 | 1.77 | 170.10 | 96.10 |
| Cycle 1 – Load 2 | 1.77 | 125.21 | 70.74 |
| Cycle 1 – Load 3 | 1.80 | 137.53 | 76.41 |
| Cycle 1 – Load 4 | 1.78 | 127.60 | 71.68 |
| **Cycle 1 - Mean** | **1.78** | **140.11** | **78.73** |
| **Cycle 1 – Std Dev** | **0.01** | **20.69** | **11.84** |
| Cycle 4 – Load 1 | 1.77 | 94.72 | 53.52 |
| Cycle 4 – Load 2 | 1.77 | 72.43 | 40.92 |
| Cycle 4 – Load 3 | 1.80 | 100.48 | 55.82 |
| Cycle 4 – Load 4 | 1.78 | 52.70 | 29.61 |
| **Cycle 4 - Mean** | **1.78** | **80.08** | **44.97** |
| **Cycle 4 – Std Dev** | **0.01** | **21.90** | **12.15** |
| Cycle 8 – Load 1 | 1.77 | 55.90 | 31.58 |
| Cycle 8 – Load 2 | 1.77 | 38.43 | 21.71 |
| Cycle 8 – Load 3 | 1.80 | 38.70 | 21.50 |
| Cycle 8 – Load 4 | 1.78 | 51.80 | 29.10 |
| **Cycle 8 - Mean** | **1.78** | **46.21** | **25.97** |
| **Cycle 8 – Std Dev** | **0.01** | **8.98** | **5.14** |
| Cycle 16 – Load 1 | 1.77 | 49.98 | 28.24 |
| Cycle 16 – Load 2 | 1.77 | 59.47 | 33.60 |
| **Cycle 16 - Mean** | **1.77** | **54.72** | **30.92** |
| **Cycle 16 – Std Dev** | **0.00** | **6.71** | **3.79** |
| Cycle 32 – Load 1 | 1.77 | 49.41 | 27.91 |
| Cycle 32 – Load 2 | 1.77 | 72.70 | 41.07 |
| **Cycle 32 - Mean** | **1.77** | **61.05** | **34.49** |
| **Cycle 32 – Std Dev** | **0.00** | **16.47** | **9.30** |
| Cycle 48 – Load 1 | 1.77 | 31.75 | 17.94 |
| Cycle 48 – Load 2 | 1.77 | 34.47 | 19.47 |
| **Cycle 48 - Mean** | **1.77** | **33.11** | **18.70** |
| **Cycle 48 – Std Dev** | **0.00** | **1.92** | **1.09** |
| **European Pod + Fabric Softener** | | | |
|  | **Load mass**  **(kg)** | **Microfiber mass**  **(mg)** | **Microfiber release**  **(ppm)*** |
| Cycle 1 – Load 1 | 1.78 | 148.30 | 83.31 |
| Cycle 1 – Load 2 | 1.78 | 154.93 | 87.04 |
| Cycle 1 – Load 3 | 1.78 | 124.45 | 69.91 |
| Cycle 1 – Load 4 | 1.79 | 162.23 | 90.63 |
| **Cycle 1 - Mean** | **1.78** | **147.48** | **82.72** |
| **Cycle 1 – Std Dev** | **0.01** | **16.37** | **9.05** |
| Cycle 4 – Load 1 | 1.78 | 48.03 | 26.99 |
| Cycle 4 – Load 2 | 1.78 | 134.05 | 75.31 |
| Cycle 4 – Load 3 | 1.78 | 84.46 | 47.45 |
| Cycle 4 – Load 4 | 1.79 | 153.88 | 85.97 |
| **Cycle 4 - Mean** | **1.78** | **105.11** | **58.93** |
| **Cycle 4 – Std Dev** | **0.01** | **47.96** | **26.78** |
| Cycle 8 – Load 1 | 1.78 | 30.26 | 17.00 |
| Cycle 8 – Load 2 | 1.78 | 118.52 | 66.58 |
| Cycle 8 – Load 3 | 1.78 | 44.37 | 24.93 |
| Cycle 8 – Load 4 | 1.79 | 67.35 | 37.62 |
| **Cycle 8 - Mean** | **1.78** | **65.12** | **36.53** |
| **Cycle 8 – Std Dev** | **0.01** | **38.74** | **21.76** |
| Cycle 16 – Load 1 | 1.78 | 54.18 | 30.44 |
| Cycle 16 – Load 2 | 1.78 | 43.70 | 24.55 |
| **Cycle 16 - Mean** | **1.78** | **48.94** | **27.49** |
| **Cycle 16 – Std Dev** | **0.00** | **7.41** | **4.16** |
| Cycle 32 – Load 1 | 1.78 | 40.34 | 22.66 |
| Cycle 32 – Load 2 | 1.78 | 42.49 | 23.87 |
| **Cycle 32 - Mean** | **1.78** | **41.41** | **23.26** |
| **Cycle 32 – Std Dev** | **0.00** | **1.52** | **0.85** |
| Cycle 48 – Load 1 | 1.78 | 44.60 | 25.06 |
| Cycle 48 – Load 2 | 1.78 | 52.43 | 29.46 |
| **Cycle 48 - Mean** | **1.78** | **48.52** | **27.26** |
| **Cycle 48 – Std Dev** | **0.00** | **5.54** | **3.11** |

***Microfiber release (ppm) = Microfiber mass (mg) / Load mass (kg)**
