# Supplementary material for: Microfiber release from real soiled consumer laundry and the impact of fabric care products and washing conditions
Source: PLoS One. 2020 Jun 5;15(6):e0233332. doi: 10.1371/journal.pone.0233332 (PMC7274375; doi:10.1371/journal.pone.0233332)
Supplement: S8 Table — U.S.A. Traditional Top Loader (T-TL) and High-Efficiency Top-Loader (HE-TL) conditions. (DOCX) [file pone.0233332.s011.docx]

**S11** **Table. Impact of fabric softener on microfiber release from polyester fleece and performance T-shirt (n = 64).** U.S.A. Traditional Top Loader (T-TL) and High-Efficiency Top-Loader (HE-TL) conditions.

| **USA Pod** | | | |
| --- | --- | --- | --- |
|  | **Load mass**  **(kg)** | **Microfiber mass**  **(mg)** | **Microfiber release (ppm)*** |
| **Fleece: T-TL** |  |  |  |
| Cycle 2 – Load 1 | 2.32 | 142.58 | 61.46 |
| Cycle 2 – Load 2 | 2.34 | 131.15 | 56.05 |
| Cycle 2 – Load 3 | 2.33 | 163.86 | 70.32 |
| Cycle 2 – Load 4 | 2.31 | 163.20 | 70.65 |
| **Cycle 2 - Mean** | **2.33** | **150.20** | **64.62** |
| **Cycle 2 – Std Dev** | **0.01** | **16.09** | **7.13** |
| Cycle 4 – Load 1 | 2.32 | 93.79 | 40.43 |
| Cycle 4 – Load 2 | 2.34 | 89.28 | 38.15 |
| Cycle 4 – Load 3 | 2.33 | 72.09 | 30.94 |
| Cycle 4 – Load 4 | 2.31 | 91.73 | 39.71 |
| **Cycle 4 - Mean** | **2.33** | **86.72** | **37.31** |
| **Cycle 4 – Std Dev** | **0.01** | **9.93** | **4.35** |
| **Fleece: HE-TL** |  |  |  |
| Cycle 2 – Load 1 | 2.36 | 88.47 | 37.49 |
| Cycle 2 – Load 2 | 2.35 | 42.21 | 17.96 |
| Cycle 2 – Load 3 | 2.34 | 35.74 | 15.27 |
| Cycle 2 – Load 4 | 2.33 | 58.04 | 24.91 |
| **Cycle 2 - Mean** | **2.35** | **56.11** | **23.91** |
| **Cycle 2 – Std Dev** | **0.01** | **23.51** | **9.92** |
| Cycle 4 – Load 1 | 2.36 | 19.24 | 8.15 |
| Cycle 4 – Load 2 | 2.35 | 35.15 | 14.96 |
| Cycle 4 – Load 3 | 2.34 | 19.04 | 8.14 |
| Cycle 4 – Load 4 | 2.33 | 17.38 | 7.46 |
| **Cycle 4 - Mean** | **2.35** | **22.70** | **9.68** |
| **Cycle 4 – Std Dev** | **0.01** | **8.34** | **3.53** |
| **T-Shirt: T-TL** |  |  |  |
| Cycle 2 – Load 1 | 1.68 | 41.21 | 24.53 |
| Cycle 2 – Load 2 | 1.68 | 58.15 | 34.61 |
| Cycle 2 – Load 3 | 1.68 | 85.75 | 51.04 |
| Cycle 2 – Load 4 | 1.70 | 71.43 | 42.02 |
| **Cycle 2 - Mean** | **1.69** | **64.13** | **38.05** |
| **Cycle 2 – Std Dev** | **0.01** | **18.99** | **11.24** |
| Cycle 4 – Load 1 | 1.68 | 41.80 | 24.88 |
| Cycle 4 – Load 2 | 1.68 | 29.86 | 17.77 |
| Cycle 4 – Load 3 | 1.68 | 49.61 | 29.53 |
| Cycle 4 – Load 4 | 1.70 | 36.11 | 21.24 |
| **Cycle 4 - Mean** | **1.69** | **39.34** | **23.36** |
| **Cycle 4 – Std Dev** | **0.01** | **8.40** | **5.03** |
| **T-Shirt: HE-TL** |  |  |  |
| Cycle 2 – Load 1 | 1.69 | 31.47 | 18.62 |
| Cycle 2 – Load 2 | 1.67 | 29.55 | 17.69 |
| Cycle 2 – Load 3 | 1.67 | 24.57 | 14.71 |
| Cycle 2 – Load 4 | 1.67 | 33.78 | 20.23 |
| **Cycle 2 - Mean** | **1.68** | **29.84** | **17.81** |
| **Cycle 2 – Std Dev** | **0.01** | **3.92** | **2.32** |
| Cycle 4 – Load 1 | 1.69 | 26.21 | 15.51 |
| Cycle 4 – Load 2 | 1.67 | 27.65 | 16.55 |
| Cycle 4 – Load 3 | 1.67 | 23.05 | 13.80 |
| Cycle 4 – Load 4 | 1.67 | 14.65 | 8.77 |
| **Cycle 4 - Mean** | **1.68** | **22.89** | **13.66** |
| **Cycle 4 – Std Dev** | **0.01** | **5.82** | **3.45** |
| **USA Pod + Fabric Softener** | | | |
|  | **Load mass**  **(kg)** | **Microfiber mass**  **(mg)** | **Microfiber release**  **(ppm)*** |
| **Fleece: T-TL** |  |  |  |
| Cycle 2 – Load 1 | 2.34 | 123.77 | 52.89 |
| Cycle 2 – Load 2 | 2.31 | 114.42 | 49.53 |
| Cycle 2 – Load 3 | 2.32 | 241.16 | 103.95 |
| Cycle 2 – Load 4 | 2.31 | 254.57 | 110.20 |
| **Cycle 2 - Mean** | **2.32** | **183.48** | **79.15** |
| **Cycle 2 – Std Dev** | **0.01** | **74.64** | **32.38** |
| Cycle 4 – Load 1 | 2.34 | 80.25 | 34.29 |
| Cycle 4 – Load 2 | 2.31 | 90.60 | 39.22 |
| Cycle 4 – Load 3 | 2.32 | 103.29 | 44.52 |
| Cycle 4 – Load 4 | 2.31 | 84.64 | 36.64 |
| **Cycle 4 - Mean** | **2.32** | **89.70** | **38.67** |
| **Cycle 4 – Std Dev** | **0.01** | **10.01** | **4.39** |
| **Fleece: HE-TL** |  |  |  |
| Cycle 2 – Load 1 | 2.34 | 65.48 | 27.98 |
| Cycle 2 – Load 2 | 2.34 | 36.22 | 15.48 |
| Cycle 2 – Load 3 | 2.35 | 57.61 | 24.52 |
| Cycle 2 – Load 4 | 2.35 | 30.93 | 13.16 |
| **Cycle 2 - Mean** | **2.35** | **47.56** | **20.28** |
| **Cycle 2 – Std Dev** | **0.01** | **16.61** | **7.09** |
| Cycle 4 – Load 1 | 2.34 | 50.59 | 21.62 |
| Cycle 4 – Load 2 | 2.34 | 19.13 | 8.17 |
| Cycle 4 – Load 3 | 2.35 | 19.20 | 8.17 |
| Cycle 4 – Load 4 | 2.35 | 29.65 | 12.62 |
| **Cycle 4 - Mean** | **2.35** | **29.64** | **12.65** |
| **Cycle 4 – Std Dev** | **0.01** | **14.81** | **6.34** |
| **T-Shirt: T-TL** |  |  |  |
| Cycle 2 – Load 1 | 1.70 | 72.79 | 42.82 |
| Cycle 2 – Load 2 | 1.70 | 67.53 | 39.72 |
| Cycle 2 – Load 3 | 1.68 | 66.13 | 39.36 |
| Cycle 2 – Load 4 | 1.70 | 82.40 | 48.47 |
| **Cycle 2 - Mean** | **1.70** | **72.21** | **42.59** |
| **Cycle 2 – Std Dev** | **0.01** | **7.37** | **4.21** |
| Cycle 4 – Load 1 | 1.70 | 39.48 | 23.22 |
| Cycle 4 – Load 2 | 1.70 | 38.16 | 22.45 |
| Cycle 4 – Load 3 | 1.68 | 25.11 | 14.95 |
| Cycle 4 – Load 4 | 1.70 | 19.37 | 11.40 |
| **Cycle 4 - Mean** | **1.70** | **30.53** | **18.00** |
| **Cycle 4 – Std Dev** | **0.01** | **9.87** | **5.77** |
| **T-Shirt: HE-TL** |  |  |  |
| Cycle 2 – Load 1 | 1.69 | 30.11 | 17.81 |
| Cycle 2 – Load 2 | 1.69 | 63.01 | 37.28 |
| Cycle 2 – Load 3 | 1.69 | 46.86 | 27.73 |
| Cycle 2 – Load 4 | 1.70 | 42.24 | 24.85 |
| **Cycle 2 - Mean** | **1.69** | **45.55** | **26.92** |
| **Cycle 2 – Std Dev** | **0.01** | **13.61** | **8.07** |
| Cycle 4 – Load 1 | 1.69 | 31.21 | 18.47 |
| Cycle 4 – Load 2 | 1.69 | 32.58 | 19.28 |
| Cycle 4 – Load 3 | 1.69 | 26.48 | 15.67 |
| Cycle 4 – Load 4 | 1.70 | 31.34 | 18.44 |
| **Cycle 4 - Mean** | **1.69** | **30.40** | **17.96** |
| **Cycle 4 – Std Dev** | **0.01** | **2.69** | **1.58** |

***Microfiber release (ppm) = Microfiber mass (mg) / Load mass (kg)**
